# Curative Gastrectomy for Advanced Gastric Cancer in a Patient with Idiopathic Multicentric Castleman Disease: A Rare Case Report

**DOI:** 10.70352/scrj.cr.25-0318

**Published:** 2025-07-01

**Authors:** Ryohei Kawabata, Yuki Ushimaru, Hisashi Hara, Tomohira Takeoka, Yumiko Yasuhara, Terukazu Yoshihara, Akihiro Kitagawa, Takashi Takeda, Hideo Tomihara, Atsushi Naito, Masahiro Murakami, Shingo Noura, Atsushi Miyamoto

**Affiliations:** 1 Department of Gastrointestinal Surgery, Sakai City Medical Center, Sakai, Osaka, Japan; 2 Department of Gastrointestinal Surgery, Osaka International Cancer Center, Osaka, Osaka, Japan; 3 Department of Pathology, Sakai City Medical Center, Sakai, Osaka, Japan

**Keywords:** idiopathic multicentric Castleman disease, gastric cancer, lymphadenopathy, curative surgery, immunosuppression

## Abstract

**INTRODUCTION:**

Idiopathic multicentric Castleman disease (iMCD) is a rare lymphoproliferative disorder characterized by systemic inflammation and chronic immunosuppression. When solid malignancies such as gastric cancer arise in patients with iMCD, perioperative management becomes particularly challenging due to nutritional decline, reactive lymphadenopathy, and elevated surgical risk.

**CASE PRESENTATION:**

A 75-year-old man with a 26-year history of suspected iMCD treated with low-dose corticosteroids presented with epigastric discomfort. Endoscopy revealed a Borrmann type 2 lesion, and biopsy confirmed poorly differentiated adenocarcinoma. CT showed mild lymphadenopathy along the lesser curvature and left gastric artery, as well as systemic involvement. Inguinal node biopsy confirmed polyclonal plasma cell proliferation consistent with iMCD. The patient also met the Asian Working Group for Sarcopenia (AWGS) criteria for severe sarcopenia. A multidisciplinary team initiated preoperative respiratory rehabilitation, nutritional support, and resistance exercise therapy. Curative distal gastrectomy with D2 lymphadenectomy was performed without complications. Histopathology revealed pT2N0M0 (pStage IB) disease. Tocilizumab was started 3 months postoperatively, and the patient remains recurrence-free at 24 months.

**CONCLUSIONS:**

This case highlights that, even in patients with long-standing iMCD and sarcopenia, carefully staged multimodal perioperative care—including accurate nodal evaluation and individualized systemic therapy—can enable successful curative surgery for advanced gastric cancer.

## INTRODUCTION

Castleman disease is a rare, non-clonal lymphoproliferative disorder characterized by systemic inflammation and generalized lymphadenopathy.^1,2)^ Among its subtypes, idiopathic multicentric Castleman disease (iMCD) is not associated with viral infection or paraneoplastic syndromes and typically follows a chronic course marked by immune dysregulation, anemia, hypoalbuminemia, and multi-site lymph node enlargement.^3–5)^

When solid malignancies such as gastric cancer occur in patients with iMCD, several clinical challenges arise.^6–8)^ Preoperative staging may be confounded by reactive lymphadenopathy, while long-term corticosteroid use and chronic inflammation can compromise nutritional status and physical function, often leading to sarcopenia, thereby increasing surgical risk.^7,9)^ Such patients require careful multidisciplinary evaluation, individualized surgical planning, and staged systemic therapy to ensure safe and effective oncologic management.^10)^

Although a few case reports have described the coexistence of Castleman disease and solid tumors, cases involving gastric cancer remain extremely rare.^6,11)^ Moreover, no previous studies have reported curative resection of gastric cancer in a patient with a pre-existing diagnosis of long-term treated iMCD.

Herein, we report the case of a patient with long-standing iMCD who underwent curative gastrectomy for advanced gastric cancer. This case highlights the importance of accurate staging, preoperative histologic evaluation of lymphadenopathy, and multidisciplinary perioperative decision-making in managing malignancy in patients with chronic inflammatory disorders.

## CASE PRESENTATION

A 75-year-old man was referred to our department with epigastric discomfort. Twenty-six years earlier, he had presented with generalized lymphadenopathy and polyclonal hypergammaglobulinemia, raising suspicion of iMCD. However, a definitive diagnosis could not be established at that time due to the inability to obtain diagnostic tissue. The patient was maintained on low-dose oral prednisolone (3 mg/day). His past medical history included herpes zoster, vertebral compression fracture, type 2 diabetes mellitus, hypothyroidism, and obstructive ventilatory impairment. Preoperative pulmonary function tests showed a forced vital capacity (FVC) of 3.27 L (102.2% predicted) and a forced expiratory volume in one second (FEV_1_) of 1.94 L (59.3% predicted).

**Fig. 1:**
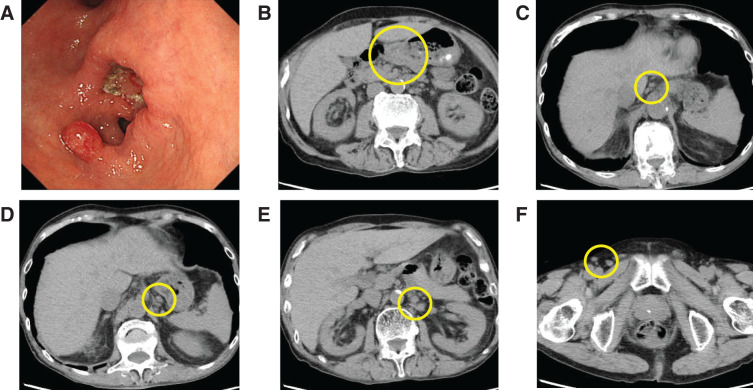
(**A**) Upper gastrointestinal endoscopy showing a circumferential Borrmann type 2 lesion along the lesser curvature of the gastric antrum. (**B**–**F**) Plain computed tomography images showing mild wall thickening in the gastric antrum and mildly enlarged lymph nodes (≤10 mm) along the lesser curvature and around the left gastric artery, as well as systemic lymphadenopathy including the para-aortic and inguinal regions.

Initial laboratory tests revealed severe anemia (hemoglobin 7.6 g/dL), hypoalbuminemia (serum albumin 1.8 g/dL), elevated C-reactive protein (CRP: 5.74 mg/dL), and mild renal impairment (serum creatinine 1.82 mg/dL). Preoperative laboratory findings are summarized in **Table 1**. Upper gastrointestinal endoscopy revealed a circumferential Borrmann type 2 lesion along the lesser curvature of the antrum (**Fig. 1A**), and biopsy confirmed poorly differentiated adenocarcinoma. Computed tomography revealed antral wall thickening (**Fig. 1B**) and mild enlargement (≤10 mm) of lymph nodes along the lesser curvature and around the left gastric artery (**Fig. 1C**, **1D**), as well as systemic lymphadenopathy involving the para-aortic region (**Fig. 1E**); and the bilateral axillary, supraclavicular, mediastinal, hilar, pelvic, and inguinal regions (**Fig. 1F**).

**Table 1:** Preoperative laboratory values at presentation

Parameter	Value	Unit	Reference range
Hematology			
RBC	2.60 × 10^6^	/μL	4.35–5.55 × 10^6^
Hemoglobin (Hb)	7.6	g/dL	13.7–16.8
Hematocrit (Ht)	23.6	%	40.7–50.1
MCV	90.8	fL	83.6–98.2
MCH	29.2	pg	27.5–33.2
MCHC	32.2	g/dL	31.7–35.3
WBC	6,840	/μL	3,300–8,600
Platelets (PLT)	27.1 × 10^4^	/μL	15.8–34.8 × 10^4^
Biochemistry			
AST	28	U/L	13–30
ALT	30	U/L	10–42
ALP	136	U/L	38–113
LDH	98	U/L	124–222
γ-GTP	14	U/L	13–64
Total bilirubin (T-Bil)	0.14	mg/dL	0.4–1.5
Albumin (Alb)	1.8	g/dL	4.1–5.1
Na	133	mEq/dL	138–145
K	4.0	mEq/dL	3.6–4.8
Cl	106	mEq/dL	101–108
BUN	40.9	mg/dL	8–20
Creatinine (Cre)	1.82	mg/dL	0.65–1.07
C-reactive protein (CRP)	5.74	mg/dL	<0.14
Glycated hemoglobin (HbA1c)	6.2	%	4.6–6.2
Coagulation			
Prothrombin time (PT)	53.9	%	70–130
PT-INR	1.34		0.85–1.15
APTT	36.5	Sec	24–32
Tumor markers			
CEA	1.8	ng/mL	<5.0
CA19-9	11.5	U/mL	<37

To evaluate the etiology of lymphadenopathy, an excisional biopsy was performed on a mildly enlarged right inguinal lymph node. Histopathological examination revealed small reactive follicles and dense interfollicular infiltration of polyclonal plasma cells, with scattered Dutcher bodies. Immunohistochemistry showed mixed kappa and lambda light chains without restriction, and staining for human herpesvirus 8 (HHV-8) was negative. These findings were consistent with iMCD.

The clinical stage of the gastric cancer was assessed as cT3N1M0 (cStage III). After multidisciplinary discussion, curative resection of the gastric cancer was prioritized over initiation of iMCD-directed therapy. The patient was also diagnosed with severe sarcopenia according to the criteria of the Asian Working Group for Sarcopenia (AWGS),^12)^ based on decreased grip strength (23.4 kg), slow gait speed (0.83 m/s), and low skeletal muscle index (SMI, 6.2 kg/m^2^). Therefore, in addition to red blood cell transfusion, we initiated nutritional intervention and resistance exercise therapy for sarcopenia. Preoperative intervention lasted approximately 4 weeks. Nutritional support consisted of outpatient dietary counseling, including intake assessment, target energy/protein guidance, and proposal of oral nutritional supplements (ONS). Exercise therapy included leaflet-based resistance training and daily walking. For chronic respiratory disease, a combination of long-acting muscarinic antagonist (LAMA) and beta2-agonist (LABA) was introduced under pulmonologist supervision. Perioperative swallowing assessment and functional conditioning were also provided by speech and physical therapists. The patient subsequently underwent robot-assisted distal gastrectomy with D2 lymphadenectomy and Billroth I reconstruction. The operation lasted 3 hours and 47 minutes with minimal blood loss. No serosal exposure of the tumor was observed intraoperatively, and mild lymph node enlargement was noted in the dissection field. Perioperative corticosteroid supplementation was administered to prevent adrenal insufficiency and inflammatory complications due to long-term steroid use.

**Fig. 2:**
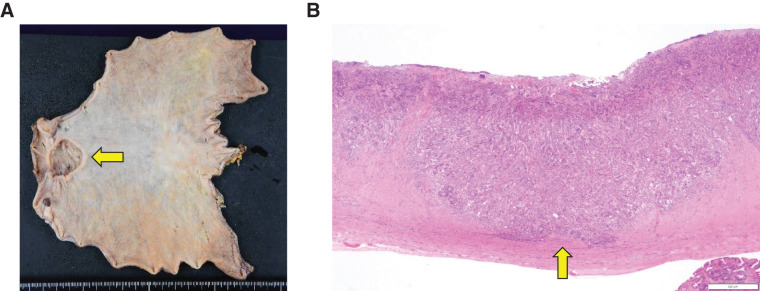
(**A**) Gross appearance of the resected gastric specimen, showing a 33 × 30 mm type 2 tumor in the antrum (arrow). (**B**) Histological examination of the tumor demonstrating a poorly differentiated adenocarcinoma with invasion into the muscularis propria (pT2).

**Fig. 3:**
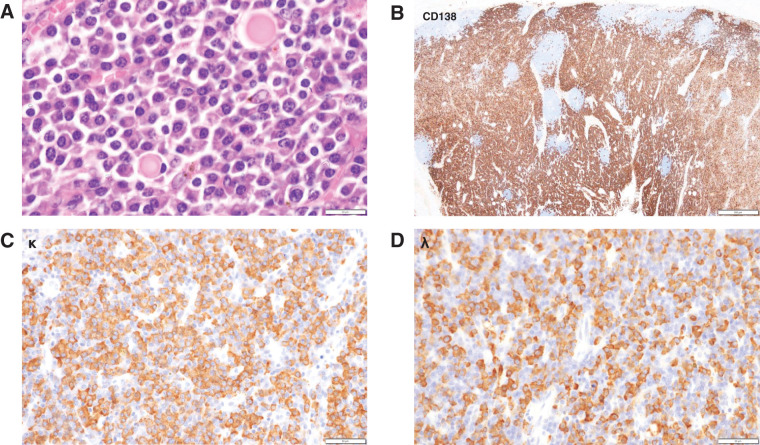
Histopathological evaluation of a resected lymph node. (**A**) Hematoxylin and eosin staining reveals dense interfollicular infiltration of plasma cells and reactive germinal centers. (**B**) Immunohistochemical staining for CD138 confirms polyclonal plasma cell proliferation. (**C**) Immunostaining for immunoglobulin κ light chain. (**D**) Immunostaining for immunoglobulin λ light chain, showing no light chain restriction.

Gross examination of the resected specimen revealed a 33 × 30 mm type 2 tumor (**Fig. 2**). Histologically, the tumor was a poorly differentiated adenocarcinoma that had invaded the muscularis propria (pT2) and showed lymphatic and venous invasion (Ly1c, V1c), but no nodal or perineural invasion. Surgical margins were negative, and the final pathological stage was pT2N0M0 (Stage IB). Resected lymph nodes showed polyclonal CD138-positive plasma cell proliferation without light-chain restriction, consistent with reactive changes related to iMCD (**Fig. 3**).

The postoperative course was uneventful, and the patient was discharged on postoperative day 9. Tocilizumab therapy was initiated 3 months after surgery, and the patient remains free of gastric cancer recurrence and iMCD flare at 24 months postoperatively.

## DISCUSSION

Idiopathic multicentric Castleman disease (iMCD) is a rare lymphoproliferative disorder characterized by IL-6-driven systemic inflammation, anemia, hypoalbuminemia, and generalized lymphadenopathy.^2–5)^ When a solid malignancy such as gastric cancer arises in the context of iMCD, surgical decision-making becomes particularly challenging due to overlapping factors including immunosuppression, nutritional deterioration, and the potential for radiologic overestimation of disease stage caused by reactive lymphadenopathy.^7,8)^

In the present case, preoperative CT demonstrated mild enlargement (up to 10 mm) of lymph nodes along the lesser curvature and around the left gastric artery, leading to a clinical diagnosis of cN1 gastric cancer based on the Japanese Classification of Gastric Carcinoma. However, given the patient’s long-standing iMCD, reactive lymphadenopathy was strongly suspected. To clarify the nature of the nodal findings, an excisional biopsy of a mildly enlarged right inguinal lymph node was performed before surgery. Histological analysis revealed reactive follicles, dense interfollicular polyclonal plasma cell infiltration, scattered Dutcher bodies, negative staining for HHV-8, and no evidence of light chain restriction, findings consistent with iMCD. This confirmed the likelihood of non-metastatic lymphadenopathy and facilitated more appropriate staging and surgical planning. Although PET-CT was initially planned for this patient to further evaluate the extent of lymphadenopathy, the examination was ultimately not performed due to concerns about insurance coverage for iMCD in Japan. According to recent consensus guidelines and the literature, PET-CT is considered useful in assessing the systemic distribution and disease activity of lymphadenopathy in iMCD, and may help distinguish between iMCD and other malignancies such as lymphoma or metastatic disease.^4,13,14)^ However, FDG uptake in iMCD is not specific and may overlap with findings seen in malignancies. Therefore, histological confirmation remains essential. In this case, an excisional lymph node biopsy was performed instead, which allowed for definitive diagnosis and facilitated appropriate treatment planning for the concurrent advanced gastric cancer.

The patient had been treated with low-dose corticosteroids for over 20 years. At the time of cancer diagnosis, he presented with severe anemia (hemoglobin 7.6 g/dL), hypoalbuminemia (serum albumin 1.8 g/dL), and elevated C-reactive protein (CRP: 5.74 mg/dL), indicating significant inflammation and nutritional decline. After multidisciplinary discussion, a decision was made to prioritize curative resection of the gastric cancer. Preoperative preparation included respiratory rehabilitation, transfusion of two units of red blood cells, perioperative steroid coverage, nutritional support and resistance exercise therapy for sarcopenia (conducted over more than 2–3 weeks), and infection control measures. Anti-IL-6 therapy was deferred until wound healing was confirmed.

Tocilizumab, a monoclonal antibody targeting the IL-6 receptor, is considered the preferred alternative to siltuximab in Japan.^2,15,16)^ However, its use in the perioperative setting requires caution due to its potential to suppress CRP responses and delay wound healing. In this case, tocilizumab was safely initiated 3 months after surgery without complication. The patient has remained free from recurrence of both gastric cancer and iMCD flare for 24 months.

According to the risk calculator provided by the National Clinical Database (NCD) of Japan,^17)^ the estimated risks for operative mortality and postoperative pneumonia in this patient were 19.1% and 19.0%, respectively. Despite these high-risk estimates, the patient experienced no perioperative complications. This favorable outcome may be attributed to a multidisciplinary approach involving preoperative respiratory rehabilitation, epidural analgesia for postoperative pain control, and perioperative steroid coverage. In addition, the patient had been diagnosed with severe sarcopenia, and preoperative nutritional support combined with resistance exercise therapy was implemented. According to the 2021 ESPEN guidelines on perioperative nutrition, preoperative nutritional intervention for at least 7–14 days is recommended for malnourished patients, even if surgery must be postponed.^18)^ Furthermore, a meta-analysis in the field of sarcopenia treatment emphasizes the benefit of combined nutritional and resistance exercise interventions in improving physical function and clinical outcomes.^19)^ Although the optimal duration of such intervention remains unclear, in this case, approximately 4 weeks of multidisciplinary prehabilitation was feasible following lymph node biopsy and before the gastrectomy. These interventions likely helped improve his physical function and contributed to a more robust perioperative recovery. These coordinated efforts likely contributed to respiratory stability, immune modulation, and enhanced recovery, even in the setting of long-term immunosuppression.

Coexistence of gastric cancer and iMCD is exceedingly rare, with most reported cases involving incidental diagnosis of Castleman disease during or after surgery.^8,11)^ To the best of our knowledge, this is the first report of curative gastrectomy with D2 lymphadenectomy in a patient with pre-existing, clinically managed iMCD. This case illustrates that with careful staging, multidisciplinary coordination, and appropriately timed systemic therapy, major oncologic surgery can be safely performed even in patients with chronic inflammatory disease.

## CONCLUSIONS

We report a rare case of advanced gastric cancer arising during the long-term clinical course of iMCD, in which curative gastrectomy was successfully performed following multidisciplinary planning. This case highlights the importance of accurate clinical staging, preoperative lymph node assessment, and coordinated perioperative management in achieving safe and effective surgical outcomes in patients with chronic inflammatory conditions such as iMCD.

## ACKNOWLEDGMENTS

We sincerely thank all medical staff involved in the care of this patient, including physicians, nurses, pharmacists, rehabilitation specialists, pathologists, and other allied health professionals. Their dedicated multidisciplinary efforts were essential for the successful management of this complex case.

## DECLARATIONS

### Funding

No funding was obtained for this study. The funding body played no role in the design of the study; collection, analysis, and interpretation of data; or writing of the manuscript.

### Authors’ contributions

RK and HH collected the clinical data and drafted the manuscript.

RK, YU, and TTakeoka participated in the clinical management of the patient.

YY performed the pathological analysis.

All authors have read and approved the final manuscript, and agree to be held accountable for all aspects of the research.

### Availability of data and materials

Not applicable.

### Ethics approval and consent to participate

This work does not require ethical considerations or approval. Informed consent to participate in this study was obtained from the patient.

### Consent for publication

Informed consent for publication was obtained from the patient.

### Competing interests

The authors have no competing interests to declare.
